# Environmental and Individual Correlates of Various Types of Physical Activity among Community-Dwelling Middle-Aged and Elderly Japanese

**DOI:** 10.3390/ijerph10052028

**Published:** 2013-05-17

**Authors:** Yoshinobu Saito, Yuko Oguma, Shigeru Inoue, Ayumi Tanaka, Yoshitaka Kobori

**Affiliations:** 1 Department of Health Promotion, Fujisawa City Health and Medical Foundation, 5527-1 Oba, Fujisawa, Kanagawa 251-0861, Japan; E-Mail: ayumi-t@fhmc.or.jp; 2 Sports Medicine Research Center, Keio University, 4-1-1 Hiyoshi, Kohoku-ku, Yokohama, Kanagawa 223-0061, Japan; E-Mail: yoguma@a7.keio.jp; 3 Graduate School of Health Management, Keio University, 4411, Endo, Fujisawa, Kanagawa 252-0816, Japan; 4 Department of Preventive Medicine and Public Health, Tokyo Medical University, 6-1-1 Shinjuku, Shinjuku-ku, Tokyo 160-8402, Japan; E-Mail: inoue@tokyo-med.ac.jp; 5 Department of Medicine, Fujisawa City Health and Medical Foundation, 5527-1 Oba, Fujisawa, Kanagawa 251-0861, Japan; E-Mail: yoshitaka-k@fhmc.or.jp

**Keywords:** leisure-time physical activity, walking, neighborhood environment, public health

## Abstract

Recent studies have suggested the importance of the neighborhood environment in determining the specific type of physical activity. However, few studies on this topic have been undertaken in Japan. This study examined the association of three types of physical activity and their associations with individual and neighborhood environmental factors among middle-aged and elderly Japanese. Participants were 2,449 adults aged 40–69 living in Fujisawa city who had undergone health checkups and responded to our survey by mail. Individual factors, the International Physical Activity Questionnaire (long form), and its environmental module acted as inputs to the study. The adjusted odds ratios (ORs) of high levels of moderate-to-vigorous intensity leisure-time physical activity (LTPA), walking for active recreation, and transportation were calculated in relation to individual and neighborhood environmental factors through multiple logistic regression models. Not working and good self-rated health were significantly associated with a higher level of each physical activity outcome. According to the adjusted ORs, higher educational attainment, higher economic status, good access to exercise facilities, and owning motor vehicles were associated with longer LTPA time. However, different sets of factors were associated with longer walking times for recreation and transportation. The results suggest that diverse individual and neighborhood environmental characteristics are associated with different physical activity outcomes. Therefore, customizing environments to become activity-friendly is necessary to increase physical activity effectively among middle-aged and elderly Japanese.

## 1. Introduction

Regular physical activity reduces the risk of mortality and the incidence of cardiovascular diseases, diabetes mellitus, and some forms of cancers [1,2,3]. However, the majority of the population in Japan and in many other countries is not physically active [2,4,5]. According to the National Health and Nutrition Survey in Japan, the average number of daily steps (measured by pedometer) has decreased in 2009 from that in 1997 (7,243 *vs.* 8,202 in men and 6,431 *vs.* 7,282 in women) [5]. Physical inactivity has been identified as the fourth leading risk factor for global mortality, with roughly 6% of global deaths being attributed to it, trailing behind only high blood pressure (13%), tobacco use (9%), and elevated blood glucose levels (6%) [3]. When we consider the fact that obesity is responsible for 5% of global mortality [3], we can appreciate why the promotion of physical activity is a public health priority.

Better understanding of the determinants of physical activity can guide effective physical activity promotion. Individual-level interventions to promote physical activity have been shown to be effective in the short term. However, such programs are often limited to a small number of participants, and the long-term effects of these interventions have not been assessed [6]. Recently, environmental factors have been highlighted in studies investigating physical activity. The ecological model emphasizes a multi-level approach (individual, social, and environmental). Studies based on this theoretical framework have revealed environmental factors related to physical activity [7,8,9,10,11], and it is expected that individual-level programs would be enhanced by a multi-level approach, which might produce long-term effects [12]. 

Physical activity is carried out in different domains, such as at home, for transportation, at work, and for leisure. Recent studies have shown that environmental factors affect the different domains of physical activity differently [9,10,11,13,14,15,16], particularly that of transportation. Important predictors of this domain of physical activity are residential density, accessibility of destinations, street connectivity, and mixed land use planning [9,10,13,17,18]. In contrast, the environmental factors that affect recreational activities are still being debated. Access to exercise facilities and aesthetics has been identified as significant predictors of leisure-time physical activities (LTPA) [9,10,13,14]. However, other studies have reported no associations between these environmental attributes and leisure-time physical activities [10,19,20].

Despite evidence for the associations between specific types and domains of physical activity and the environment, research in this area has been mostly limited to Western countries, particularly to the United States and Australia [11], with only a few studies conducted in Japan [13,14,21]. Thus, evidence from countries where the environment, culture, and physical activity patterns differ from those of Western countries is valuable. Indeed, evidence from Japan could support or refute the generalizations of previous studies conducted in Western countries and reveal new findings regarding associations between the environment and physical activity. Furthermore, only a few studies conducted in Japan have included the elderly population [14,22] even though the relevance of environmental factors increases with age. Data from this target population will be highly useful towards the development of physical activity interventions in Japan.

In this cross-sectional study, we attempt to address these issues by studying a random community sample of adults aged 40–69 in Fujisawa city, Japan, with LTPA and walking as the dependent variables. We aim to examine the individual and environmental correlates of physical activity and its components, including moderate-to-vigorous LTPA, walking for recreation, and walking for transportation.

## 2. Methods

### 2.1. Participants and Data Collection

We recruited Japanese adults aged 40–69 in Fujisawa city who were National Health Insurance beneficiaries. The subjects had undergone the Specific Health Checkup, which focuses on metabolic syndrome, in 2009, and had responded to a mailed cross-sectional survey in 2010. Fujisawa city (population: 403,912 as of 1 March 2010) is located about 40 km southwest of Tokyo.

From approximately 20,000 participants who underwent the Specific Health Checkup among the 56,000 National Health Insurance beneficiaries, 4,165 (2,076 men and 2,089 women) were randomly selected by using the postal pin code and stratification by sex, with a sample size of 100 from each postal number area. Of the 4,165 subjects identified, 2,610 (62.7%) responded to the survey. Ultimately, valid data were obtained from 2,449 participants (final response rate: 58.8%).

The study received prior approval from the Keio University Ethics Committee, Graduate School of Health Management. All participants gave written informed consent before answering the questionnaire.

### 2.2. Assessment of Physical Activity

Moderate-to-vigorous LTPA excluding walking, recreational walking, transportation walking, and sitting time were assessed with relevant items from the Japanese version of the International Physical Activity Questionnaire (IPAQ)—Long Form [23,24]. The IPAQ long form assesses physical activity by asking for details about the specific types of activities undertaken in specific domains, *i.e.*, transportation, domestic chores and gardening (yard), and leisure-time. This instrument is designed for use by adults (age range: 15–69 years). The reliability and validity of this questionnaire has been demonstrated in 12 countries, including Japan [23]. Test-retest reliability for total physical activity of the Japanese version of IPAQ was adequate (Spearman’s *ρ* = 0.93), and the criterion validity for total physical activity assessed against the accelerometer was comparable to other survey measures (Spearman’s *ρ* = 0.36).

Participants reported the number of days and average hours and minutes per day in which they were engaged in moderate and vigorous LTPA, and walking for recreation and transportation for at least 10 min at a time in a typical week. Weekly minutes of each type of physical activity were calculated according to the IPAQ scoring manual [25]. The following values were used for the analysis of IPAQ data: moderate LTPA (e.g., bicycling at a regular pace, swimming at a regular pace, or playing doubles tennis) = 4.0 metabolic equivalents (METs), vigorous LTPA (e.g., aerobics, running, fast bicycling, or fast swimming) = 8.0 METs, and walking for recreation and transportation (e.g., to travel to and from work, to do errands, or to go from place to place) = 3.3 METs. 

### 2.3. Assessment of Perceived Neighborhood Environment

To assess perceived neighborhood environments, we administered the environmental module of the IPAQ (IPAQ-E) [14,26,27,28]. The IPAQ-E consists of 17 questions: seven core items, four recommended items, and six optional items. In this study, we used 11 items (the core and recommended items). 

Our questions assess residential density (the main types of houses in neighborhoods), access to shops, public transport, presence of sidewalks and bicycle lanes, access to exercise facilities, crime safety, traffic safety, social environment (seeing people being active), aesthetics (the qualities of a neighborhood that encourage participants to walk), and number of motor vehicles in the participant’s household. These questions refer to a neighborhood environment where a person could walk within 10 to 15 min from their residence. The question pertaining to residential density has five response options: (1) detached single-family housing; (2) townhouses, row houses, apartments, or condominiums of 2–3 stories; (3) a mix of single-family residences and townhouses, row houses, apartments, or condominiums; (4) apartments or condominiums of 4–12 stories; and (5) apartments or condominiums of more than 12 stories. One question concerns the number of motor vehicles owned by the household. The other 9 questions have a 4-point Likert response scale (strongly disagree, somewhat disagree, somewhat agree, and strongly agree).

This questionnaire has been used previously and has good reliability [26,28]. We used the Japanese version of the IPAQ-E, which is available online [29]. Spearman correlation coefficients of the Japanese version of the IPAQ-E were between 0.79 and 0.99 for the 11 items. Kappa statistics also showed good reliability and ranged from 0.63 to 0.97 [26].

### 2.4. Individual Variables

Information on participants’ gender and age were obtained from the database of the Specific Health Checkup. Information regarding years of education, employment status, household economic status, marital status, number of children in the household, self-rated health, and the presence of severe pain caused by orthopedic disorders was obtained through self-reports.

### 2.5. Statistical Analyses

Statistical analyses were performed in accordance with methods used in previous studies [30]. To examine the association between individual and neighborhood environment (independent variables) and moderate-to-vigorous LTPA, recreational walking, and transport-related walking (dependent variables), odds ratios for active participants were calculated through logistic regression models. We had confirmed in advance that all independent variables were not correlated strongly with each other. All individual and environmental variables were dichotomized as shown in Table 1.

**Table 1:** Distribution of individual and neighborhood environmental attributes entered into the final logistic regression model (n = 1,940).

Characteristics		n	%
[Individual attributes]			
	Gender		
	Men	943	48.6
Women		997	51.4
Age (years)			
	40–49	153	7.9
	50–59	279	14.4
	60–64	527	27.2
	65–69	981	50.6
Education(years)			
	≤12	1,050	54.1
	>12	890	45.9
Working status			
	Not working	1,088	56.1
	Working	852	43.9
Household economic status			
	Bad/very bad	868	44.7
	Very good/good	1,072	55.3
Marital status			
	Never married	137	7.1
	Married	1,572	81
	Divorced/Widowed	231	11.9
Presence of children in the household			
	Yes	121	6.2
	No	1,819	93.8
Self-rated health			
	Poor/very poor	360	18.6
	Very good/good	1,580	81.4
Experience strong pain due to orthopedic disorders			
	Yes	95	4.9
	No	1,845	95.1
[Neighborhood environmental attributes]			
Residential density			
	Low	1,096	56.5
	High	844	43.5
Access to shops			
	Poor	585	30.2
	Good	1,355	69.8
Access to public transport			
	Poor	89	4.6
	Good	1,851	95.4
Presence of sidewalks			
	No	760	39.2
	Yes	1,180	60.8
Presence of bicycle lanes			
	No	1,236	63.7
	Yes	704	36.3
Access to exercise facilities			
	Poor	717	37.0
	Good	1,223	63.0
Crime safety			
	Not safe	760	39.2
	Safe	1,180	60.8
Traffic safety			
	Not safe	723	37.3
	Safe	1,217	62.7
Social environment			
	Poor	490	25.3
	Good	1,450	74.7
Aesthetics			
	Poor	840	43.3
	Good	1,100	56.7
Motor vehicles owned			
	One or more	1,638	84.4
	None	302	15.6

Note: Percentages do not always add up to 100% because of rounding errors.

Pearson’s chi-square tests were used to examine the association between moderate-to-vigorous LTPA, recreational walking, transportation walking, and each independent variable. All independent variables with *p* ≤ 0.10 were included in the following logistic regression analyses to guard against potential type II errors and to avoid overfitting. Multivariate logistic regression modeling involved the forced entry of gender, age (four categories), and education (two categories) in the first block. In the second block, a stepwise backward elimination procedure was used to identify the independent variables that were significantly related to moderate-to-vigorous LTPA, recreational walking, and transportation walking, respectively. Removal testing is based on the probability of the likelihood-ratio statistic, which is based on maximum partial likelihood estimates. We set the probability for stepwise as *p* < 0.05 for inclusion and *p* < 0.10 for exclusion.

Environmental variables were converted into dichotomous variables for analysis, as mentioned previously. “Detached single-family residences” were categorized as low residential density while the other choices were categorized as high residential density. The responses to the number of motor vehicles owned were categorized as “none” and “one or more.” Responses to the other questions were classified into two categories: “agree” (strongly agree and somewhat agree) and “disagree” (somewhat disagree and strongly disagree). 

For each of the three physical activity variables, participants were dichotomized according to the median. Due to missing values, the logistic regression model of moderate-to-vigorous LTPA included only 1,940 participants while the model of recreational walking and transportation walking included 1,937 and 1,896 participants respectively. In the final model, variables that obtained *p* < 0.05 In the Wald test were considered to be statistically significant. All analyses were conducted through PASW version 18.0 for Windows (SPSS Inc., Tokyo, Japan).

## 3. Results

### 3.1. Participant Characteristics

Table 1 shows the frequency distributions for the independent variables associated with the three types of physical activity (*p* ≤ 0.10) (*i.e.*, moderate-to-vigorous LTPA, recreational walking, and transport-related walking), which were then entered into the logistic regression models. The study sample included slightly more women (51.4%) than men. The average age (standard deviation: SD) was 62.4 (6.8) years. About 54% of the participants had received high school education or less.

Among those who responded to the survey (n = 2,400), 1,641 (68%) did not engage in moderate-to-vigorous LTPA excluding walking while 759 (32%) exercised for leisure. Among those who answered the question regarding recreational walking (n = 2,400), 1,471 (61%) walked for recreation. Among those who answered the question about transport-related walking (n = 2,327), 1,764 (76%) walked for transportation. The median (range) values of the three types of physical activity were as follows: moderate-to-vigorous LTPA: 0 (0–3,300) min/week; recreational walking: 60 (0–2,520) min/week; transportation walking: 90 (0–1,890) min/week

### 3.2. Individual Factors

Table 2 shows the adjusted odds ratios (ORs) for individual and neighborhood environmental variables of participants who engaged in moderate-to-vigorous LTPA, recreational walking, and transport-related walking compared to their non-active counterparts. 

Among the individual factors, not working and good self-rated health increased the odds of engaging in all three types of physical activity. Presence of children in the household decreased the odds of engaging in all three types of physical activity, although only that for transport-related walking was significant.

Household economic status increased the odds of engaging in moderate-to-vigorous LTPA but decreased the odds of engaging in transport-related walking. Being younger decreased the odds of engaging in the two types of walking (*p* for trend < 0.01). 

**Table 2:** Adjusted odds ratios for individual and neighborhood environmental characteristics for moderate-to-vigorous intensity LTPA (n = 1,940) and recreational (n = 1,937)/transport-related (n = 1,896) walking.

		Moderate-to-Vigorous Intensity LTPA			Recreational Walking			Transport-related Walking		
		OR ^a^	(95% CI)	*P-*value	OR ^a^	(95% CI)	*P-*value	OR ^a^	(95% CI)	*P-*value
Gender										
	Men	1.00			1.00			1.00		
	Women	1.00	(0.82–1.23)	0.987	0.67	(0.55–0.81)	<0.001	1.12	(0.92–1.36)	0.243
Age (years)										
	65–69	1.00			1.00			1.00		
	60–64	0.89	(0.71–1.13)	0.339	0.87	(0.70–1.08)	0.205	0.79	(0.63–0.98)	0.034
	50–59	0.63	(0.46–0.87)	0.005	0.56	(0.42–0.76)	<0.001	0.77	(0.57–1.03)	0.076
	40–49	1.05	(0.70–1.58)	0.809	0.35	(0.23–0.54)	<0.001	0.73	(0.50–1.06)	0.097
	*P* for trend			0.193			<0.001			0.009
Education (years)										
	≤12	1.00			1.00			1.00		
	>12	1.65	(1.34–2.02)	<0.001	0.85	(0.70–1.03)	0.088	1.01	(0.83–1.23)	0.900
Working status										
	Not working	1.00			1.00			1.00		
	Working	0.69	(0.56–0.85)	0.001	0.52	(0.42–0.63)	<0.001	0.79	(0.64–0.96)	0.018
Household economic status										
	Bad/very bad	1.00			-			1.00		
	Very good/good	1.40	(1.14–1.72)	0.001	-			0.83	(0.68–1.01)	0.057
	No	1.00			1.00			1.00		
	Yes	0.67	(0.43–1.03)	0.071	0.69	(0.45–1.04)	0.079	0.64	(0.43–0.96)	0.029
Self-rated health										
	Poor/very poor	1.00			1.00			1.00		
	Very good/good	2.47	(1.84–3.31)	<0.001	1.75	(1.37–2.25)	<0.001	1.53	(1.20–1.95)	0.001
	Yes	-			1.00			-		
	No	-			1.57	(1.00–2.49)	0.052	-		
Residential density										
	Low	1.00			-					
	High	0.84	(0.69–1.03)	0.090	-					
Access to exercise facilities										
	Poor	1.00			-			-		
	Good	1.27	(1.03–1.56)	0.022	-			-		
Motor vehicles owned										
	One or more	1.00			-			1.00		
	None	0.65	(0.49–0.87)	0.003	-			2.16	(1.66–2.82)	<0.001
Social environment										
	Poor	-			1.00			-		
	Good	-			1.41	(1.12–1.76)	0.003	-		
Aesthetics										
	Poor	-			1.00			-		
	Good	-			1.34	(1.10–1.63)	0.004	-		
Access to shops										
	Poor	-			-			1.00		
	Good	-			-			1.41	(1.15–1.74)	0.001
Presence of sidewalks										
	Poor	-			-			1.00		
	Good	-			-			1.26	(1.04–1.53)	0.018

LTPA: leisure-time physical activity excluding walking; OR, odds ratios; CI, confidence interval; ^a^ Active participants were defined as those engaging in moderate-to-vigorous LTPA for ≥10 min/week, walking for recreation for ≥70 min/week, and walking for transportation for ≥100 min/week. Multivariate logistic regression modeling involved the forced entry of gender, age (4 categories), and education (2 categories) in the first block. In the second block, a stepwise backward elimination procedure was used to identify the independent variables that were significantly related to moderate-to-vigorous LTPA, recreational walking, and transportation walking, respectively.

Being younger, except for being in the 40 to 49 age bracket, decreased the odds of engaging in moderate-to-vigorous LTPA. Higher educational attainment increased the odds of engaging in moderate-to-vigorous LTPA. Being a woman decreased the odds of engaging in recreational walking. Experiencing strong pain due to orthopedic disorders also decreased the odds of engaging in recreational walking.

### 3.3. Environmental Factors

Among environmental factors, good access to exercise facilities and owning motor vehicles increased the odds of engaging in moderate-to-vigorous LTPA other than walking. The social environment and aesthetics increased the odds of engaging in recreational walking. On the other hand, good access to shops and presence of sidewalks increased the odds of engaging in transport-related walking while owning motor vehicles decreased the odds. Marital status, access to public transport, presence of bicycle lanes, crime safety, and traffic safety were not included in the final fitted model.

## 4. Discussion

This cross-sectional study revealed that among community-dwelling middle-aged and elderly Japanese, individual and perceived neighborhood environmental characteristics were associated with physical activity, and that these associations varied by the type (*i.e.*, domain and intensity) of physical activity. Consistent with previous studies, there were differences in the association between environmental characteristics and the three types of physical activities [9,10,11,13,14,15]. We found that moderate-to-vigorous LTPA was associated with three sociodemographic (educational attainment, working status, and economic status) and two environmental (access to exercise facilities and number of motor vehicles) characteristics and self-rated health. Recreational walking was associated with three sociodemographic (gender, age, and working status) and two environmental (social environment and aesthetics) characteristics and self-rated health. Walking for transportation was associated with three sociodemographic (age, working status, and presence of children in the household) and three environmental (number of motor vehicles, access to shops, and presence of sidewalks) characteristics and self-rated health.

The model showed a positive association between not working and good self-rated health and all three types of physical activity. Our results showed that being female decreased the odds of engaging in recreational walking. The model also showed a positive association between age and walking. More than half of our participants were aged 65 or older, and were unemployed. It is therefore probable that older and retired people, especially men, can devote more time towards walking. Furthermore, from a physiological point of view, walking intensity could be adjusted according to one’s level of physical fitness. Since walking can generally be taken up as a form of exercise by anyone and does not require access to expensive facilities, it is more likely to be popular with elderly men.

Previous research in other countries has found socio economic status and self-rated health to be significant correlates of physical activity [31,32]. Moderate-to-vigorous LTPA was associated with socioeconomic status in terms of higher educational attainment and higher economic status. These results suggest that LTPA involves considerably higher spending compared to walking (probably manifested through buying sporting goods, payment for facility charges, *etc.*). Thus, people with higher family incomes usually have better access to physical activity facilities and opportunities, and consequently, had better health (which is due also, in part, to better access to health care resources). 

We also found that not experiencing severe pain due to orthopedic disorders was marginally associated with recreational walking. Furthermore, a similar association was also observed for LTPA and transport-related walking. However, because about 95% of the participants in our study did not report experiencing severe pain due to orthopedic disorders, we may have to discount this association.

A position statement by the Heart Foundation’s National Physical Activity Advisory Committee in Australia has summarized recent studies of neighborhood environmental characteristics and walking in adults [33]. The statement highlighted four characteristics that were consistently correlated with transport-related walking—population density, proximity of destinations (including shops and public transport), mixed land use planning, and street connectivity (the “walkability index”). It also indicated three characteristics correlated with recreational walking: access to exercise facilities, pedestrian infrastructure, and aesthetics. Our results showed that some of these factors are consistently associated with specific types of physical activity among middle-aged and elderly individuals in Fujisawa city. The results of our study were consistent with the committee’s statement. As the committee’s statement suggested, multilevel intervention targeting a specific population and behavior is important. Our results suggest that the proximity of destinations and sidewalks are important factors for increasing transport-related walking when designing and redesigning residential environments. Giles-Corti *et al.* provided evidence for long-term changes in transport and recreational-walking behaviors according to changes in the availability and diversity of local transport and recreational destinations and demonstrated the potential of local infrastructure in supporting health-enhancing behaviors [34].

In addition, changes in the micro-level environment, such as the social environment (seeing people being active), and aesthetics (the qualities of a neighborhood that encourage participants to walk) could improve the perception of the neighborhood environment, which could increase recreational walking [35].

Despite accumulating evidence for the association between physical activity and the environment, research has been mostly limited to walking or general physical activity [13,14,21,26,36,37,38]. To our knowledge, no study in Japan has focused on LTPA excluding walking and environmental characteristics. We found that moderate-to-vigorous LTPA was related to two environmental characteristics (access to exercise facilities and number of motor vehicles). However, according to our study, these environmental characteristics are not related to walking per se. Thus, the present study demonstrates that different perceived environmental characteristics are associated with different physical activity domains.

An interesting finding from this study was that owning motor vehicles was associated with longer moderate-to-vigorous LTPA time, but not with walking for transportation or recreation. It suggests that participants use motor vehicles to travel to and from the exercise facilities, and as a conveyance for sporting equipment when they perform a sport. In contrast, not owning motor vehicles was associated with longer transportation-related walking time. This result is consistent with that of previous research [14]. The relationship between motor vehicle access and transportation-related walking suggests a car-dependent lifestyle among this population; that is, people with cars walked less.

Studies conducted in Western countries have identified access to exercise facilities and aesthetics as significant predictors of LTPA [9,10,13,14]. In Japan, Hanibuchi *et al.* showed, in a research on people aged 65 years or older, that the population density and presence of parks or green spaces evaluated by the geographic information system were positively associated with the frequency of engaging in sports activities (e.g., ground golf, “gateball” [Japanese croquet], walking, jogging, and physical exercise) [22]. These results are generally consistent with the results of the present study. Few studies that focused on the target population analyzed the data according to domains of physical activity. Our data showed that access to shops and presence of sidewalks had positive associations with walking for transportation. Compact urban developments might be a solution to promote physical activity in middle-aged and elderly Japanese. It is thus necessary for future studies to consider such details in order to accumulate information useful for promoting physical activity in this population.

It is also important to mention the limitations of this study. First, due to the cross-sectional design, we were unable to examine causality. Thus, longitudinal research is needed to elucidate the direction of some of these relationships. Second, both environmental and walking measures were based on self-reports. Although both IPAQ and IPAQ-E are internationally standardized measurement tools [27,28], studies using objective measures, such as accelerometers for physical activity and a geographic information system for environmental evaluation, are needed to supplement these subjective measures [21,39]. Third, this study examined National Health Insurance beneficiaries aged between 40 and 69, who had undergone the Specific Health Checkup. In Japan, the National Health Insurance is designed for people who are not eligible for an employment-based health insurance program. Therefore, participants of this study may also differ from other citizens in terms of socioeconomic status. Careful consideration is therefore needed if one wishes to generalize the results of this study. However, in Fujisawa city, National Health Insurance beneficiaries account for about 20% and 60% of citizens aged 40–59 years and 60–69 years respectively. About 80% of the participants in this study are 60–69 years old, which increases the relevance of the results of this study. In addition, because this study did not include older adults aged 70 or above, further investigations are needed for this population.

## 5. Conclusions

The aim of this study was to examine the individual and environmental correlates of physical activity and its components, including moderate-to-vigorous LTPA, walking for recreation, and walking for transportation among middle-aged and elderly Japanese. Higher educational attainment, not working, higher economic status, good self-rated health, good access to exercise facilities, and owning motor vehicles were associated with longer LTPA time. However, different sets of factors were associated with longer walking times for recreation and transportation. The present study provides new evidence on physical activity and its environmental correlates in Japan. These results indicate that diverse individual and environmental characteristics are associated with different physical activity outcomes. Based on our findings, we believe that customizing environments to become activity-friendly is necessary to increase the level of physical activity effectively among middle-aged and elderly Japanese.
